# Supplementary material for: Estimation of the Chelating Ability of an Extract from Aronia melanocarpa L. Berries and Its Main Polyphenolic Ingredients Towards Ions of Zinc and Copper
Source: Molecules. 2020 Mar 26;25(7):1507. doi: 10.3390/molecules25071507 (PMC7180758; doi:10.3390/molecules25071507)
Supplement: Supplementary file 1 [file molecules-25-01507-s001.pdf]

molecules

Supplementary data

Estimation of the Chelating Ability of an Extract from *Aronia melanocarpa* L. Berries and Its Main Polyphenolic Ingredients Towards Ions of Zinc and Copper

Sylwia Borowska [1], Jakub W. Strawa [2], Michał Tomczyk [2], and Małgorzata M. Brzóska [1,*]

[1] Department of Toxicology, Medical University of Bialystok, Adama Mickiewicza 2C, 15-222 Bialystok, Poland; sylwiaborowska86@tlen.pl (S.B.); malgorzata.brzoska@umb.edu.pl (M.M.B.)
[2] Department of Pharmacognosy, Medical University of Bialystok, Adama Mickiewicza 2A, 15-230 Bialystok, Poland; jakub.strawa@umb.edu.pl (J.W.S.); michal.tomczyk@umb.edu.pl (M.T.)
* Correspondence: malgorzata.brzoska@umb.edu.pl (M.M.B.); Tel.: +48-85-7485604

Table S1. The changes in zinc (Zn) and copper (Cu) metabolism after the administration of the 0.1% extract from the berries of *Aronia melanocarpa* L. (AE) to the female Wistar rats for up to 24 months. [1]

**Parameter**	**3 months**		**10 months**		**17 months**		**24 months**	
	**Zn**	**Cu**	**Zn**	**Cu**	**Zn**	**Cu**	**Zn**	**Cu**
Apparent absorption (%)	NS	NS	↓ 16%	↓ 24%	NS	NS	NS	NS
Retention in the body (%)	NS	NS	↓ 16%	↓ 24%	NS	NS	NS	NS
Faecal excretion (mg/24 h)	NS	NS	↑ 24%	↑ 25%	NS	NS	NS	NS
Urinary excretion (μg/24 h)	NS	NS	NS	NS	↑ 72%	NS	NS	NS
Concentrations in particular organs, tissues, and serum								
Liver (μg/g)	NS	NS	NS	NS	NS	NS	NS	NS
Kidney (μg/g)	NS	NS	NS	NS	NS	↓ 10%	NS	NS
Spleen (μg/g)	NS	↓ 11%	NS	NS	NS	NS	NS	NS
Brain (μg/g)	NS	NS	NS	NS	NS	NS	↑ 10%	NS
Heart (μg/g)	↑ 15%	↑ 35%	NS	NS	NS	NS	NS	NS
Stomach (μg/g)	↓ 9%	↓ 21%	NS	NS	NS	NS	NS	NS
Bone tissue at the femoral diaphysis (μg/g)	NS	NS	NS	NS	NS	NS	NS	NS
Bone tissue at the femoral distal epiphysis (μg/g)	NS	NS	NS	NS	NS	↑ 35%	NS	NS
Femoral muscle (μg/g)	NS	NS	NS	NS	NS	NS	↓ 16%	NS
Duodenum (μg/g)	NS	NS	NS	↓ 9%	↓ 13%	↓ 8%	NS	NS
Serum (μg/mL)	NS	↓ 19%	NS	NS	NS	NS	NS	NS

[1] Detailed data have been reported [13]; NS, statistically nonsignificant compared with the control group; ↑, increase; ↓, decrease.

Table S2. The effect of divalent ions of zinc (Zn^{2+}) and copper (Cu^{2+}) at the studied concentrations on wavelength of absorption of the extract from *Aronia melanocarpa* L. berries (AE) at the concentrations of 0.05% and 0.1% and 0.1% ethylenediaminetetraacetic acid (EDTA) at pH 2, 5.5, and 8.

AE or EDTA (wavelengths) [nm]		Zn^{2+}			Cu^{2+}		
		**0.01 mM**	**0.1 mM**	**3 mM**	**0.01 mM**	**0.05 mM**	**0.5 mM**
**pH 2**							
**0.05% AE**							
UV	283	283	283	283	283	283	283
Vis	527	527	527	527	527	527	527
**0.1% AE**							
UV	283	283	283	283	283	283	283
Vis	527	527	527	527	527	527	527
**0.1% EDTA**							
UV	219	261	261	261	261	261	261
Vis	-	-	-	-	745	745	745
**pH 5.5**							
**0.05% AE**							
UV	283	283	283	283	283	283	283
Vis	527	538	538	579	583	583	583
**0.1% AE**							
UV	283	283	283	283	283	283	287
Vis	527	538	538	579	527	583	583
**0.1% EDTA**							
UV	219	261	261	261	261	261	261
Vis	-	-	-	-	745	745	745
**pH 8**							
**0.05% AE**							
UV	283	283	283	283	283	283	283
Vis	527	527	527	527	527	527	527
**0.1% AE**							
UV	283	283	283	283	283	283	283
Vis	527	527	527	527	527	527	527
**0.1% EDTA**							
UV	219	261	261	261	261	261	261
Vis	-	-	-	-	745	745	745

UV, ultraviolet spectroscopy; Vis, visible spectroscopy

Table S3. The effect of divalent ions of zinc (Zn^{2+}) and copper (Cu^{2+}) at the studied concentrations on wavelength of absorption of the main polyphenolic compounds present in the 0.1% extract from *Aronia melanocarpa* L. berries (AE) at pH 5.5. [2]

Polyphenols (wavelengths) [nm]		Zn^{2+} 0.01 mM	Zn^{2+} 0.1 mM	Zn^{2+} 3 mM	Cu^{2+} 0.01 mM	Cu^{2+} 0.05 mM	Cu^{2+} 0.5 mM
				**0.008% C3G**			
UV	282	281	281	281	282	282	u.v.
Vis	528	538 (+10) *	538 (+10)	585 (+57)	585 (+57)	585 (+57)	585 (+57)
				**0.002% Q**			
UV	255	255	255	255	255	255	255
Vis	355	378 (+23)	379 (+24)	380 (+25)	378 (+23)	378 (+23)	378 (+23)
				**0.002% K**			
UV	265	264	264	265	265	256	256
Vis	340	349 (+9)	350 (+10)	350 (+10)	340	339	339
				**0.007% CA**			
UV	219	218	218	218	219	219	219
Vis	336	329	329	329	328	326	328
				**0.007% NCA**			
UV	219	218	218	218	219	219	219
Vis	336	329	329	329	328	328	328
				**0.013% (+)-catechin**			
UV	282	282	282	282	282	282	282
Vis	387	387	387	387	387	387	387
				**0.013% (-)-epicatechin**			
UV	282	282	282	282	282	282	282
Vis	390	390	390	390	390	390	390

[2] The influence of Zn^{2+} and Cu^{2+} at the studied concentrations on wavelength of absorption of particular polyphenolic compounds present in the 0.1% AE at pH 2 and 8 (lack of complexation) has been presented in Table S4 and Table S5, respectively.

UV, ultraviolet spectroscopy; Vis, visible spectroscopy; C3G, cyanidin 3-*O*-*β*-galactoside; Q, quercetin; K, kaempferol; CA, chlorogenic acid; NCA, neochlorogenic acid; u.v., unreadable value.

* Values in parentheses represent the shift of the maximum absorption noted after the addition of the solutions containing Zn^{2+} or Cu^{2+} to the solutions of polyphenolic compounds.

Table S4. The effect of divalent ions of zinc (Zn^{2+}) and copper (Cu^{2+}) at the studied concentrations on wavelength of absorption of polyphenolic compounds present in the 0.1% extract from *Aronia melanocarpa* L. berries (AE) at pH 2.

Polyphenols (wavelengths) [nm]		Zn^{2+}			Cu^{2+}		
		**0.01 mM**	**0.1 mM**	**3 mM**	**0.01 mM**	**0.05 mM**	**0.5 mM**
**0.008% C3G**							
UV	282	282	282	282	282	282	282
Vis	528	528	528	528	528	528	528
**0.002% Q**							
UV	255	255	255	255	255	255	255
Vis	349	349	349	349	349	349	349
**0.002% K**							
UV	265	265	265	265	265	265	265
Vis	372	372	372	372	372	372	372
**0.007% CA**							
UV	219	219	219	219	219	219	219
Vis	336	329	329	329	328	326	328
**0.007% NCA**							
UV	219	218	218	218	219	219	219
Vis	336	329	329	329	328	328	328
**0.013% (+)-catechin**							
UV	282	282	282	282	282	282	282
Vis	387	387	387	387	387	387	387
**0.013% (-)-epicatechin**							
UV	282	282	282	282	282	282	282
Vis	390	390	390	390	390	390	390

UV, ultraviolet spectroscopy; Vis, visible spectroscopy; C3G, cyanidin 3-*O*-*β*-galactoside; Q, quercetin; K, kaempferol; CA, chlorogenic acid; NCA, neochlorogenic acid.

.

Table S5. The effect of divalent ions of zinc (Zn^{2+}) and copper (Cu^{2+}) at the studied concentrations on wavelength of absorption of polyphenolic compounds present in the 0.1% extract from *Aronia melanocarpa* L. berries (AE) at pH 8.

Polyphenols (wavelengths) [nm]		Zn^{2+}			Cu^{2+}		
		**0.01 mM**	**0.1 mM**	**3 mM**	**0.01 mM**	**0.05 mM**	**0.5 mM**
**0.008% C3G**							
UV	282	282	282	282	282	282	282
Vis	528	528	528	528	528	528	528
**0.002% Q**							
UV	255	255	255	255	255	255	255
Vis	349	349	349	349	349	349	349
**0.002% K**							
UV	265	265	265	265	265	265	265
Vis	372	372	372	372	372	372	372
**0.007% CA**							
UV	219	219	219	219	219	219	219
Vis	336	329	329	329	328	326	328
**0.007% NCA**							
UV	219	218	218	218	219	219	219
Vis	336	329	329	329	328	328	328
**0.013% (+)-catechin**							
UV	282	282	282	282	282	282	282
Vis	387	387	387	387	387	387	387
**0.013% (-)-epicatechin**							
UV	282	282	282	282	282	282	282
Vis	390	390	390	390	390	390	390

UV, ultraviolet spectroscopy; Vis, visible spectroscopy; C3G, cyanidin 3-*O*-*β*-galactoside; Q, quercetin; K, kaempferol; CA, chlorogenic acid; NCA, neochlorogenic acid.

Table S6. The effect of divalent ions of zinc (Zn^{2+}) and copper (Cu^{2+}) at the studied concentrations on wavelength of absorption of ethylenediaminetetraacetic acid (EDTA) at pH 5.5.

EDTA (wavelengths) [nm]		Zn^{2+}			Cu^{2+}		
		**0.01 mM**	**0.1 mM**	**3 mM**	**0.01 mM**	**0.05 mM**	**0.5 mM**
				**0.05% EDTA**			
UV	219	261	261	261	-	-	-
Vis	356	-	-	-	745	745	745
				**0.013% EDTA**			
UV	219	261	261	261	-	-	-
Vis	356	-	-	-	745	745	745
				**0.008% EDTA**			
UV	219	261	261	261	-	-	-
Vis	356	-	-	-	745	745	745
				**0.007% EDTA**			
UV	219	265	265	265	-	-	-
Vis	356	-	-	-	745	745	745
				**0.002% EDTA**			
UV	219	265	265	265	-	-	-
Vis	356	-	-	-	745	745	745

UV, ultraviolet spectroscopy; Vis, visible spectroscopy

Supplementary Material—Assessment of the stability of the extract from the berries of *Aronia melanocarpa* L. (AE)

Methodology

The assessment of the stability of the AE was carried out using a Specord 200 spectrophotometer (Analytik Jena, Germany). Ultraviolet (UV) spectra within the range of 190–1100 nm were recorded. Spectrum correction was performed for methanol:water solutions (7:3, v/v) at pH values of 2, 5.5, and 8. The degree of acidification was obtained by adding the appropriate amount of HCl and NaOH solution. A 0.1% AE solution with three pH values was used to assess stability. Measurements were made immediately after preparation (1 h) and 24, 48, and 72 h after preparation of the solution.

Results and Discussion

The UV spectra of 0.1% AE at pH 2, 5.5, and 8 after preparation (1 h) and after 24, 48, and 72 h, presented in Figures S1–S3, show that this solution was stable at all pH during the first 24 h after preparation.

The critical region of the UV spectrum of the analyzed extract falls within the 340–640 nm wavelength range. The deflection range around 400 nm is a typical area for the band I spectrum of flavonoid compounds and band II for anthocyanins. The range above 400 nm is the band of anthocyanins, which corresponds to the spectrum of the reference substance used in the experiment—cyanidin 3-*O*-*β*-galactoside (C3G). The measurements carried out indicate a small variability of the spectrum in an acidic environment and a gradual decrease in its intensity with an increase in pH and time since preparation of the solution. This result has its justification in the chemical structure of anthocyanins and their transformations in an alkaline environment.

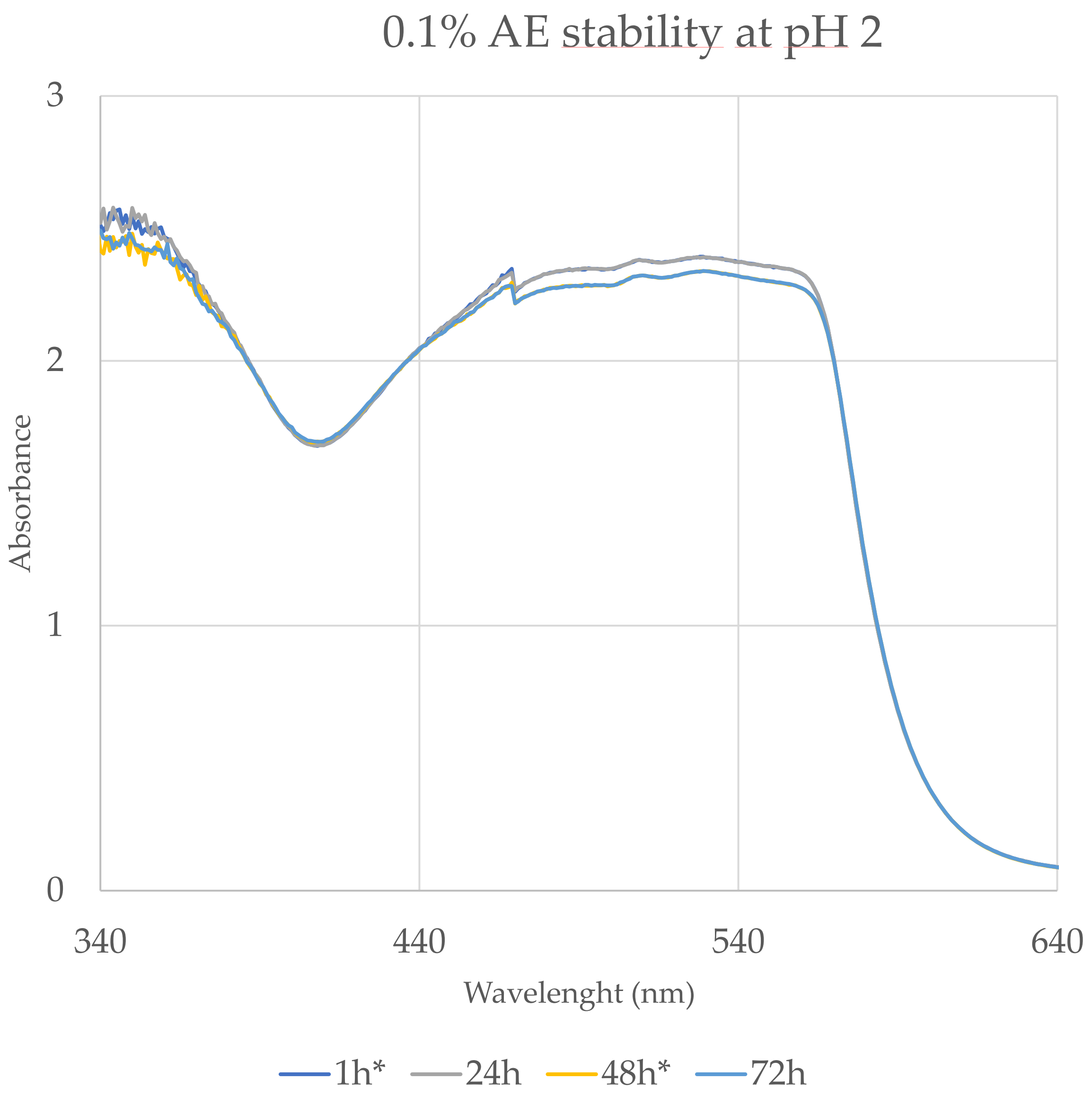

* spectrum recorded after 24 h and 72 h overlapping spectrum recorded after preparation and after 48h, respectively.

Figure S1. Ultraviolet (UV) spectrum of 0.1% extract from the berries of *Aronia melanocarpa* L. (AE) at pH 2 after preparation (1 h) and after 24, 48, and 72 h.

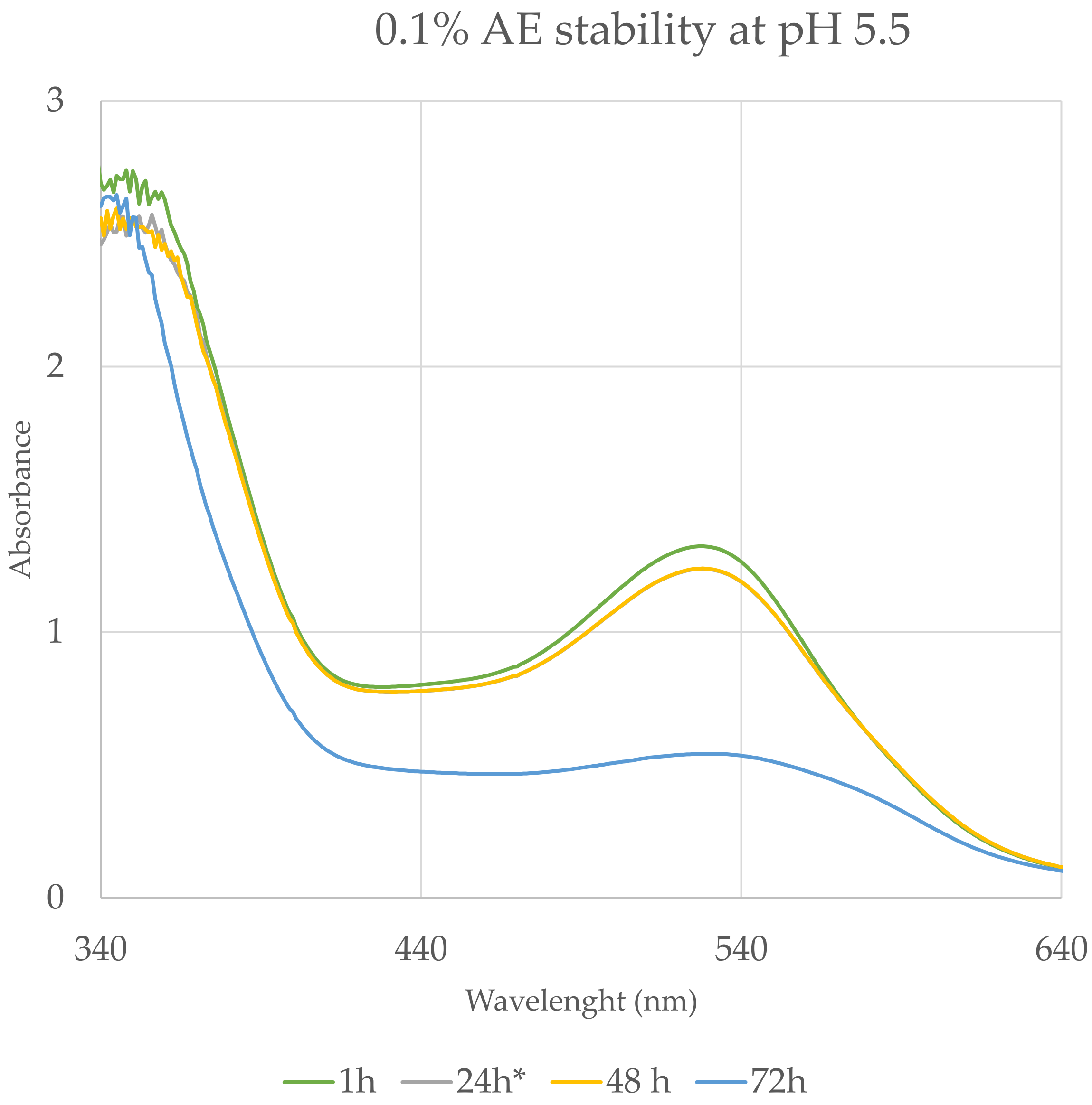

* spectrum recorded after preparation overlapping spectrum after 24h.

Figure S2. Ultraviolet (UV) spectrum of 0.05% extract from the berries of *Aronia melanocarpa* L. (AE) at pH 5.5 after preparation (1 h) and after 24, 48, and 72 h.

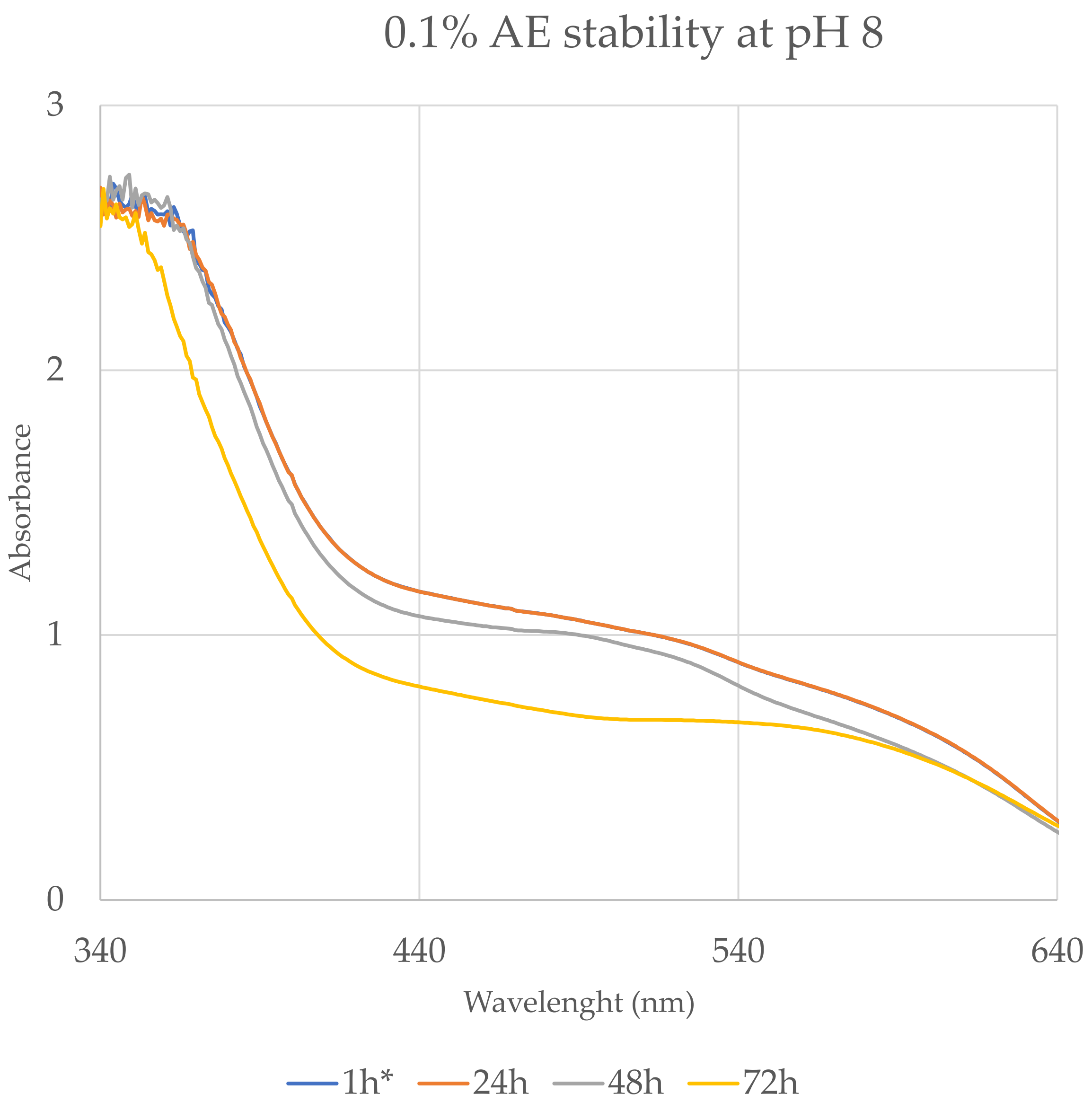

* spectrum recorded after 24 h overlapping spectrum recorded after preparation.

Figure S3. Ultraviolet (UV) spectrum of 0.05% extract from the berries of *Aronia melanocarpa* L. (AE) at pH 8 after preparation (1 h) and after 24, 48, and 72 h.
